# Contact infection of infectious disease onboard a cruise ship

**DOI:** 10.1038/srep38790

**Published:** 2016-12-08

**Authors:** Nan Zhang, Ruosong Miao, Hong Huang, Emily Y. Y. Chan

**Affiliations:** 1 Institute of Public Safety Research, Department of Engineering Physics, Tsinghua University, Beijing, China; 2 School of Energy and Power Engineering, Huangzhong University of Science and Technology, Wuhan, China; 3 JC School of Public Health and Primary Care, The Chinese University of Hong Kong, Hong Kong SAR, China

## Abstract

Cruise tourism has become more popular. Long-term personal contact, complex population flows, a lack of medical care facilities, and defective infrastructure aboard most cruise ships is likely to result in the ship becoming an incubator for infectious diseases. In this paper, we use a cruise ship as a research scenario. Taking into consideration personal behavior, the nature and transfer route of the virus across different surfaces, virus reproduction, and disinfection, we studied contact infection of infectious disease on a cruise ship. Using gastroenteritis caused by the norovirus as an example, we analyzed the characteristics of infectious disease propagation based on simulation results under different conditions. We found hand washing are the most important factors affecting virus propagation and passenger infection. It also decides either the total number of virus microorganisms or the virus distribution in different functional areas. The transfer rate between different surfaces is a key factor influencing the concentricity of the virus. A high transfer rate leads to high concentricity. In addition, the risk of getting infected is effectively reduced when the disinfection frequency is above a certain threshold. The efficiency of disinfection of functional areas is determined by total virus number and total contact times of surfaces.

With rapid population growth and economic development, convenient public transport, and high pressure of people, the number of tourists is increasing. China has the biggest population in the world and this populace takes more than 2 billion domestic tourism trips every year^1^. Since tourist destinations experience frequent large influxes of people, the risk of emergencies in these areas should receive more attention.

In recent years, because tourists preferred natural resources, uncrowded areas, easy travel, and new places, cruise tourism has become more popular^2^. In 2009, more than 13.4 million passengers embarked on a cruise ship^3^. Long-term personal contact, complex population flows, a lack of medical care facilities, and defective infrastructure aboard most cruise ships is likely to result in the ship becoming an incubator for infectious diseases. Outbreaks of infectious diseases have been frequently reported aboard cruise liners. In May 2009, 1970 passengers and more than 730 crew members were infected with the pandemic (H1N1) virus or the influenza A (H3N2) virus^4^ on cruise ships. Between February 25 and April 14, 2006, 14 rash illnesses were detected on a cruise from North America to the Caribbean^5^. In 2002, 29 outbreaks of acute gastroenteritis on cruise ships were reported by the Vessel Sanitation Program (VSP)^6^. Due to frequent outbreaks of infectious disease on board cruise ships, effective solutions to reduce the risk of epidemics are needed.

Most infectious diseases propagate via air-borne, water-borne, food-borne, vector-borne routes, as well as contact borne routes^7^. On a cruise ship, because of complex and frequent passenger movement, direct and indirect personal contact, the food-borne and water-borne infectious diseases that rely on contact transmission, should be given greater attention. For example, one ship’s infirmary reported that 84 out of 2318 passengers had gastroenteritis symptoms during a 7-day cruise in November, 2002^8^. The norovirus, which causes more than 50 percent of the gastroenteritis cases, usually breaks out on board cruise ships^9^,^10^. Because of its prevalence, we have selected it to be used as the example in this paper. Research related to infectious diseases spread by contact transmission usually uses historical data mining methods^11^, food sample analysis^12^, investigation by qu**e**stionnaire^13^, etc. Influencing factors such as sex, age, and whether the person is a crew-member or a passenger were considered. The change over time of the vertical patient distribution between decks and the number infected could be obtained^14^. All the research mentioned here mainly considers the influence of the nature of the virus and passenger behavior while usually ignoring other objective influencing factors of a cruise ship such as the inner structure, attributes of the contact surfaces, virus transfer between surfaces, and disinfection.

In this paper, we analyzed the impacts of different functional areas such as restrooms, aisles, seats, retail counters, restaurants, stair rails, and the handrails around the sightseeing deck, on norovirus gastroenteritis propagation. Taking into consideration personal behavior, the nature and transfer route of the virus across different surfaces, virus reproduction, disinfection by crews, and influences resulting from the different position of functional areas, we analyzed norovirus propagation on a cruise ship. From this analysis we provide some optimized suggestions focusing on functional area distribution, characteristics of contact surfaces, personal behavior, and disinfection on board to reduce the risk of norovirus infection.

## Methods and Parameters

### Functional areas

**Figure 1:**
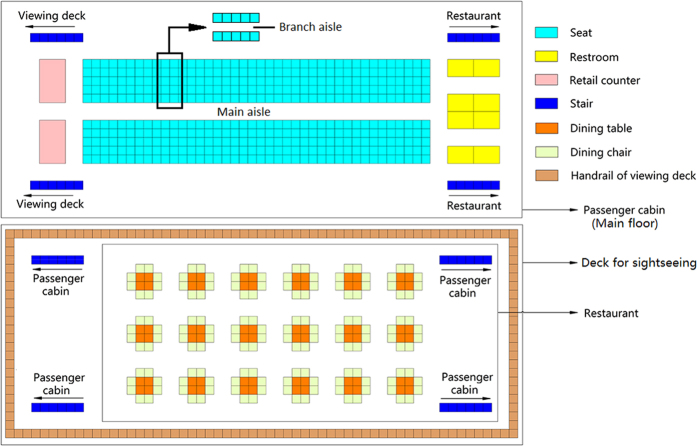
Distribution of functional areas and contact surfaces.

A cruise ship typically has many functional areas such as restrooms, seats, restaurants, etc. Passengers will go to these areas and touch surfaces at different frequencies. In this paper, we considered 6 different functional areas and 8 types of contact surfaces. The 7 functional areas are seats, restrooms, retail counters, aisle, restaurant, stair rails, and handrails around the sightseeing deck. The 8 contact surfaces include the top surface of seats, the inner and outer doorknobs of restrooms, retail counters, handrails of stairs and the sightseeing deck, and surfaces of dining tables and chairs. All surfaces are divided into multiple grids of 0.4 m × 0.4 m. Every grid is regarded as an independent contact surface. Figure 1 shows the distribution of functional areas and contact surfaces. Among them, restrooms, seats and retail counters which are included in the passenger cabin are located in the main floor. There are 400 seats which are divided into 40 rows (10 seats per row). The main aisle is set to be down the middle of all the seats. There are 8 restrooms at the right side and 2 retail counters at the left side. Passengers can get to the restaurant via the right stairs and go to the sightseeing deck via the left stairs. The restaurant is on the lower floor. There are 18 tables and 144 chairs. The deck for sightseeing is on the top floor.

The 7 functional areas are introduced below:

Seats: on a short journey aboard a cruise ship, all passengers have a seat. Because of the nature of a ship’s motion, passengers have to steady themselves by holding the top of the seats when they move from a branch aisle to the main aisle and on to other functional areas. Therefore, the top surfaces of seats are prone to be touched by passengers, especially those near the main aisle.

Restroom: norovirus easily appears in restrooms because infected passengers usually spread the virus accidentally. In this paper, a restroom is treated as the source of infection. Passengers might be unintentionally in contact with the norovirus from vomit or stools in the restroom. The restroom is the critical area for norovirus propagation on a cruise ship. There are many public facilities inside the restroom such as door handles, toilet seat, and washbasins. Passengers usually use these facilities in a regular sequence. In this paper, we regard all facilities inside a restroom as one facility which is represented by the inner door handle. For outside the restroom, the outer door handle is considered to be a contact surface.

Retail counter: the retail section is a necessary functional area on cruise ships^15^ where passengers can buy food and drink. A retail counter is a public zone that is frequently touched by both passengers and staff. This implies that a retail counter is an important surface for virus reproduction and transmission. Under actual conditions, passengers go to the retail area during specific times.

Aisle: aisles exist everywhere on cruise ships. As shown in Fig. 1, the main aisle runs between two columns of seats, and the branch aisle is sandwiched between each row of seats. Due to frequent passenger traffic in the main aisle, the seats near the main aisle are frequently touched, and we should pay attention to this area.

Stairs: on a cruise ship, different functional areas are often connected by stairs. In Fig. 1, the stairs on the left lead to the sightseeing deck, and the stairs on the right go to the restaurant. Stairs located on the deck and in the restaurant lead to the main floor. In this paper, six grids are used for each stair area, the total number of stair grids is 48 (6 × 8).

Restaurant: passengers frequently eat meals in the restaurant during meal times. Figure 1 shows 18 dining tables in the restaurant, with 8 chairs allocated to each table. We divide a table into four grids with a chair regarded as one grid. There are therefore 216 contact surfaces in the restaurant through which to spread the virus.

Sightseeing deck: passengers usually enjoy the scenery on the sightseeing deck. Handrails surround the deck for safety. As shown in Fig. 1, 58 grids make up the long side and 23 grids the short side.

There is a total of 844 grids (independent contact surfaces) in the study area (400 for seats, 2 for retail counters, 8 for inner doorknobs of restrooms, 8 for outer doorknobs of restrooms, 48 for stair handrail, 72 for dining tables, 144 for dining chairs, and 162 for the handrail of the sightseeing deck).

### Passenger behavior

**Figure 2:**
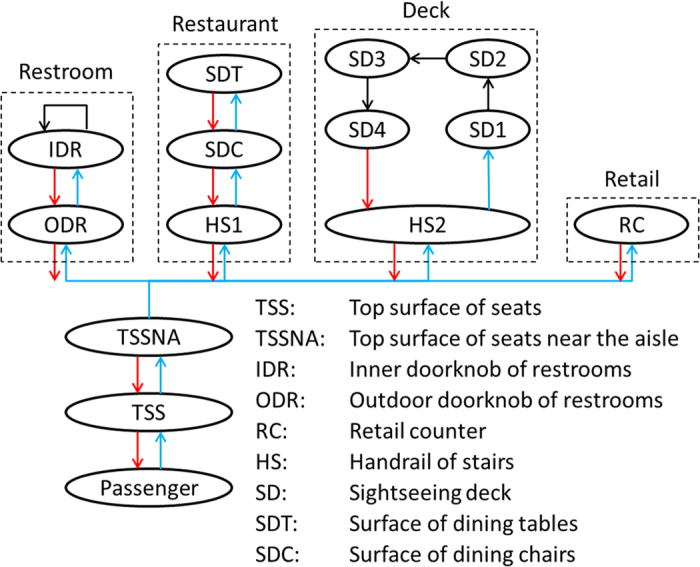
Passenger behavior in different functional areas.

Passenger behavior directly affects the distribution and transmission of a virus on board cruise ships. In this paper we define passenger behavior in different functional areas including using the toilet, eating, sightseeing, and buying. As shown in Fig. 2, different surfaces are touched as passengers move to different functional areas.

Passengers & seats: the majority of tourists travel in groups. An investigation of the tourism market from one Chinese city showed that touring with a family accounted for 54 percent of all tourists while touring with friends accounted for 31 percent^16^. The remaining 15% travel alone. On a cruise ship, passengers who are in the same group will be located in adjacent seats. Because of the motion of a big cruise ship on the seas, passengers often have to hold onto seats near the branch aisle when they move from their seat to the main aisle. They then hold the seats near the main aisle when they go to other functional areas and again on their return. In our simulation we have 2 hypotheses:

H_1_. Because practical difficulties of contact monitoring of passengers on cruise ship^17^, the probability of passengers holding onto seats when they are moving is assumed to be 100%.

H_2_. Three members are regarded as a family group and 2 to 4 members as a group of friends.

Passengers & restrooms: restroom is a critical place for passengers not only in virus infection, but also in virus disinfection. Handwashing is one of the most efficient ways to remove the norovirus on the hand^18^,^19^. When passengers want to use the restroom, they will choose the nearest vacant one (they will have to wait if all restrooms are occupied). On entering the toilet, passengers will touch the outer door handle and close the door by rotating the inner door handle. On exiting, some passengers will wash their hands, and some will not. Finally, passengers will return to their seats following the same procedures they did on the way to the restroom. The World Toilet Organization showed that people go to toilet 6 to 8 times per day (http://worldtoilet.org/). The probability of a passenger going to the restroom is therefore considered to be 1/160 every minute. According to the statistical data from different scenarios, the probability of a person washing their hands is anything between 70 and 90 percent^20^,^21^,^22^. For our purposes we set the probability of hand washing at 80 percent (the average value). Moreover, the efficacy of hand washing (percentage of the virus killed) is set to 72.5 percent based on hand washing experiments using common soap^23^. In this part, 3 hypotheses should be followed:

H_3_. One out of every seven passengers who visits the restroom will have a bowel movement.

H_4_. Defecation time is randomly distributed between 8 and 12 minutes, and urination duration is set to be 2 minutes.

Passengers & retail: the procedure followed by passengers going to a retail area is very similar to that of going to the restroom. There are 2 hypotheses for this activity:

H_5_. The probability of passengers going to a retail point per minute is set to 1/180.

H_6_. The time that passengers stay at the retail point is set to be 1 minute.

Passengers & restaurants: in a restaurant, passengers in the same group will eat at the same time^24^. They will choose a table which has the most vacant chairs. Otherwise, they will wait for an appropriate table. There are 2 hypotheses for this scenario:

H_7_. Because the majority of restaurants on a cruise ship sell fast food, the time taken by passengers to have a meal is assumed to be 25 minutes.

H_8_. According to the data set in general simulation, lunch time is usually set from 11 a.m. to 1 p.m.^25^. In this study, Initial time is 8 a.m., therefore, all passengers can get to a restaurant and have a meal taking 180 to 300 minutes.

Passengers & sightseeing deck: big cruise ships always have a big sightseeing deck. Passengers who want to enjoy the scenery can reach this deck via some stairs. In this study we assume the sightseeing deck is rectangular and obey these three hypotheses:

H_9_. The average frequency for passengers going to the sightseeing deck is once per 2 hours, and the probability of tourists going to the deck is 1/120 times per minute.

H_10_. Passengers will enjoy the scenery in all four directions and they will touch the four contact surfaces from four different sides.

H_11_. They will remain on deck for 12 minutes (3 minutes per side).

In this study, some value of parameters we mentioned above came from the references and other value we hypothesized from H_1_ to H_11_. Based all the hypotheses, the results can only reflect the norovirus propagation in the cruise ship whose structure is the same with setting in our study.

### Virus

#### Virus transfer between the different contact surfaces

In this paper we have used norovirus gastroenteritis as an example to simulate and analyze infectious disease propagation through contact^26^. Since this study focuses on contact infection, the transfer rate between different surfaces is a very important parameter. Two identical surfaces should carry the same volume of virus if they experience sufficient contact. However, the transfer rate between different surfaces is influenced by many factors such as the surface chemistry, roughness, and topology^27^. There are many different surface materials on a cruise ship, such as skin (hand), stainless (door handle, handrail), wood or plastic (dining table and chair), leather or cloth (surface of seats). Because there are so many influencing factors, it is difficult to determine the virus transfer rate between the different surfaces. In this paper, we use the following hypothesis as the virus transfer rate:

H_12_. Because contact duration is limited we assume a virus transfer rate of 0.1. In other words, 10 percent of the virus will be transferred from the higher-density surface to the lower-density surface (as shown in Equation 1).


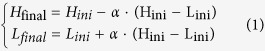


where H_final_, H_ini_, L_final_, and L_ini_ represent the final and initial colony of virus on the surfaces with higher and lower virus densities, respectively. α denotes the virus transfer rate among different surfaces (α = 0.1 is used in this study).

### Reproduction

Virus not only can transfer among different surfaces but also reproduce on the same surface following the reproduction of cells they board. Because the nature of Escherichia coli is similar to the host cell of norovirus, we used Escherichia coli as a substitute to study the reproduction. Research has shown that after 1459 minutes, a colony of Escherichia coli can increase from 10^4^ to 10^8^ under 1 atmosphere of pressure at 20 °C. When the temperature rises to 30 °C, after 490 minutes, the colony of Escherichia coli can increase from 10^5^ to 10^9^ 28. In this study, standard conditions (1 atmosphere pressure & 25 °C) are used. According to the experiments, the reproduction rate of Escherichia coli was monotonically increasing with a temperature increase from 20 to 30 °C, and accords with Eq. 2 29 (In this study, the environmental impacts on virus reproduction is ignored).


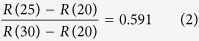


where R(20), R(25), and R(30) expresses the virus reproduction rate at a temperature of 20, 25 and 30 degrees respectively.

Combining the two previous studies mentioned above, the virus reproduction rate under standard conditions could be calculated using Eq. 3.


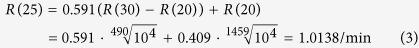


### Virus infection

The norovirus is very infectious, usually entering the body with food or water. According to previous research, people may be infected if they ingest more than 100 norovirus microorganisms^30^. For this study we conservatively hypothesize that passengers will get infected if they ingest more than 1000 norovirus microorganisms. For patients who have gastroenteritis, additional virus might be produced from vomit or stools when they are using the toilet in the restroom. Therefore, in the simulation, we assume twice the number of virus microorganisms are produced after a patient has used the toilet.

At the beginning of the simulation, we assume one person is infected with the norovirus. After going to the toilet, we assume that this person’s hand is unintentionally tainted with 3000 virus microorganisms. This time is set to be the initial time from which the norovirus started to spread on board the cruise ship.

### Disinfection

In addition to the functional areas, personal behavior, and reproduction and transmission of the virus, we also studied the impact of disinfection on virus propagation. Disinfection is a critical method of controlling or even preventing contact infection. Thorough disinfection of restroom facilities such as toilet seats, flush buttons, and door handles is very important in the prevention of infectious disease propagation on board cruise ships^31^. Due to the difficulty of disinfecting all dining chairs and top surfaces of seats that are far away from the main aisle, we simplified the disinfection of each functional area as described below. The surfaces requiring disinfection include the inner and outer door handles of restrooms, the top surfaces of seats near the main aisle, dining tables, handrails of stairs, handrails on the sightseeing deck, and retail counters. Crews on cruise ships periodically disinfect these surfaces. In the simulation, the disinfection efficiency was assumed to be 72.5%, which is the same as for hand washing.

In order to analyze virus propagation under different conditions, we assume standard values to act as the control group. Considering all the influencing factors mentioned above, the value of each factor in the control group is shown in Table 1.

## Results

### Virus propagation on a cruise ship under control conditions

**Figure 3:**
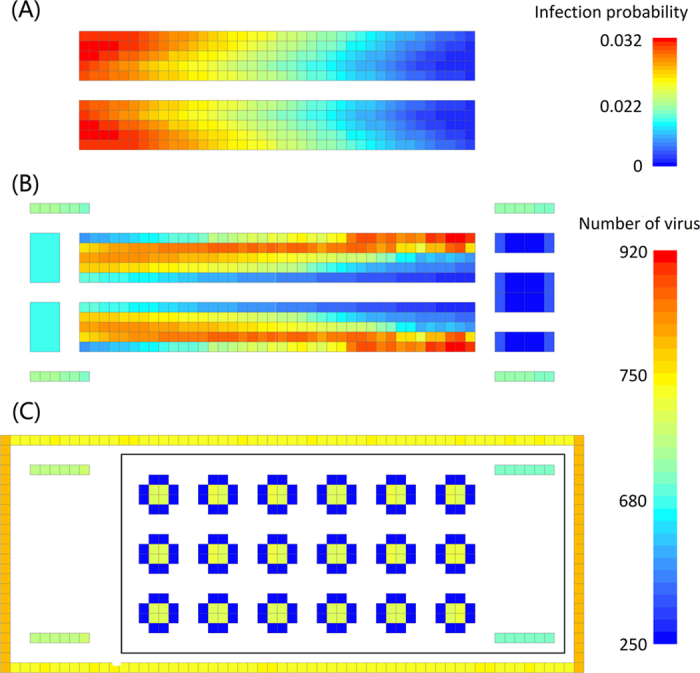
Norovirus propagation on the cruise ship under control conditions over a period of 480 minutes (8 hours). (**A**) Infection rate of passengers; (**B**) Norovirus distribution in the main floor; (**C**) Norovirus distribution in the restaurant and on the sightseeing deck.

Figure 3 shows the norovirus propagation on a cruise ship for the control conditions given in Table 1. The simulation time is set to 480 minutes (8 hours). Figure 3(A) shows the infection rate of norovirus in the cruise ship with all 400 passengers. We found that passengers on the right with seats near the restroom have a lower infection rate than passengers on the left. Because the probability and efficiency of hand washing is high (80 percent and 72.5 percent, respectively), restrooms are regarded as disinfection zones. The majority of passengers leaving the restroom are very clean (carrying a low volume of virus). After leaving the restroom, when these passengers touch other surfaces, the virus will transfer from these surfaces to their hands. Passengers who are situated closer to the restroom have a lower volume of virus. Retail counters and the stairs up to the sightseeing deck are located on the left of the main floor. These functional areas are far from the restroom and so the virus cannot be diluted easily. Therefore, the distribution of passengers getting infected accords with the characteristics of ‘the left is higher than the right’. As the incubation period of the acute gastroenteritis caused by norovirus is 12–48 hours^32^, these infected passengers would be found after 1 or 2 days. In this condition, 2.3 percent of passengers will get infected after 8 hours. Some actual situation of short-term norovirus propagation shows that the infection rate of passengers is generally not more than 5%. For example, on the cruise ship from Florida to Caribbean in 2002, 70 (4%) passengers and two (0.2%) crew members reported acute gastroenteritis (AGE) within 24 hours of embarkation^32^. On the cruise ship from Vancouver, Canada to Alaska coast in 2004, 1.24% and 4.21% (25 and 85 out of 2018 passengers) of passengers got gastrointestinal illness in the 2^nd^ and 3^rd^ day, respectively^33^. All these actual data reflect the reliability of the results from our simulation.

Figure 3(B) shows the virus distribution in the main floor. On the right, the seats near the main aisle and the restroom have low-density virus, while the seats near the wall have a high volume of the virus. On the left, the seats near the main aisle and the wall have a low volume of virus, and the left three middle seats carry a high volume of virus. Because all moving passengers will touch the seats near the main aisle, the volume of virus on these seats will be reduced. With the disinfection of the restrooms, the virus distribution is shown in Fig. 3(B). In addition, the volume of virus on the outer door handles of the restroom is higher than it is on the inner door handles. Handrails of stairs to the sightseeing deck have a higher virus density than the handrails of the stairs to the restaurant.

Figure 3(C) shows the virus distribution on the sightseeing deck and in the restaurant. The restaurant had no virus microorganisms at the start of the simulation. At meal times, passengers are viewed as infection sources. The probability of passengers touching the dining tables is much higher than the probability of touching the dining chairs, so the volume of virus on the tables is higher than it is on the chairs. On the sightseeing deck, passengers enjoy the scenery in four directions. The left and right handrails are shorter than the top and bottom handrails, and the dilution of the long handrail is greater than the short handrail. Therefore, the average volume of virus on the top and bottom handrails in the diagram is lower than it is for the left and right handrails. Moreover, the sightseeing deck has more virus microorganisms than the restaurant. The reason is that passengers only go to the restaurant once within the 8 hour period, so the virus cannot be sufficiently spread.

In general, comparing Fig. 3(B) and (C), the seats and the sightseeing deck are the areas with the highest risk for virus propagation.

### Impact of the environmental setting on virus propagation

**Figure 4:**
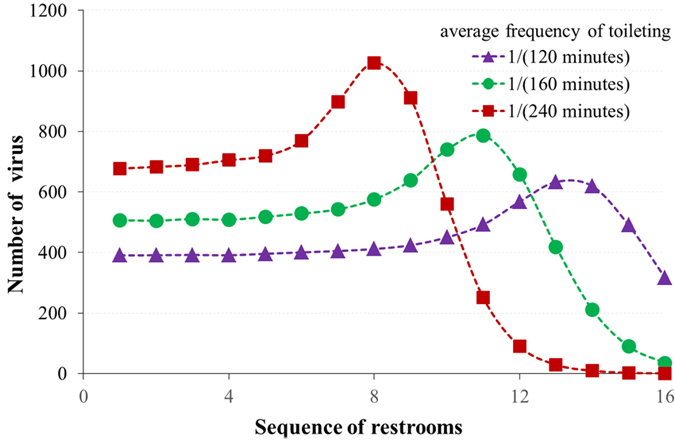
Virus distribution on outer door handles of restrooms with different usage frequencies.

Figure 4 shows the virus distribution on the outer door handles of all the available restrooms, together with the frequency at which they are used. There are 16 restrooms whose sequence is numbered according with their distance from the main aisle (NO. 1 is the nearest restroom while NO. 16 is the furthest one). The restrooms experiencing the most traffic also have the highest increase in the volume of the virus. The restrooms furthest from the main aisle have low volumes of the virus because of infrequent use. Although the restrooms near the main aisle are frequently used, more passengers will dilute the virus. From the simulation data we can see that the restrooms used between 20 and 30 times have the highest risk. Crews on cruise ships could disinfect the restrooms after they have been used a certain number of times, to maximize disinfection efficacy.

**Figure 5:**
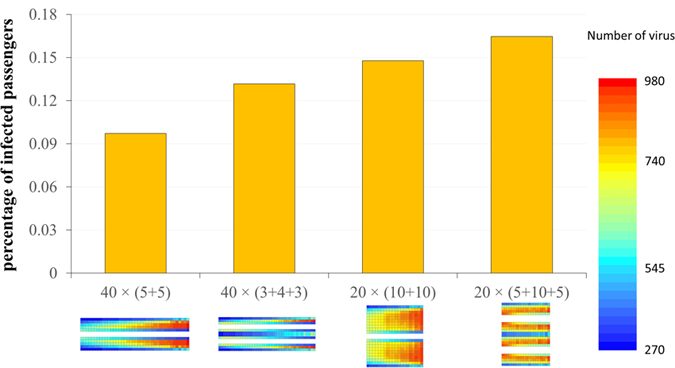
Percentage of infected passengers with different seat distributions.

Figure 5 shows the percentages of infected passengers according to the position of their seats. When the seat distribution changes from ‘40 × 10’ to ‘20 × 20’, the percentage of infected passengers will increase. For the 40 × 10 configuration, a passenger will touch other seats 21.9 times on average when going to the restroom. This is higher than the 20 × 20 configuration (13.5 times). Decreasing the contact time with seats means that less virus is transferred to the seats and therefore more virus remains on the passengers. Therefore, when the seat distribution changes from ‘40 × (5 + 5)’ to ‘40 × (3 + 4 + 3)’, the percentage of infected passengers will also increase because of the lower contact times. In the four conditions mentioned above, when the seat distribution is ‘40 × (5 + 5)’, average 38.9 passengers will be norovirus-positive after 8 hours exposure. And approaching 65.8 passengers will be norovirus-positive when the seat distribution is changed to ‘20 × (5 + 10 + 5)’.

### Virus propagation under different hand washing conditions

**Figure 6:**
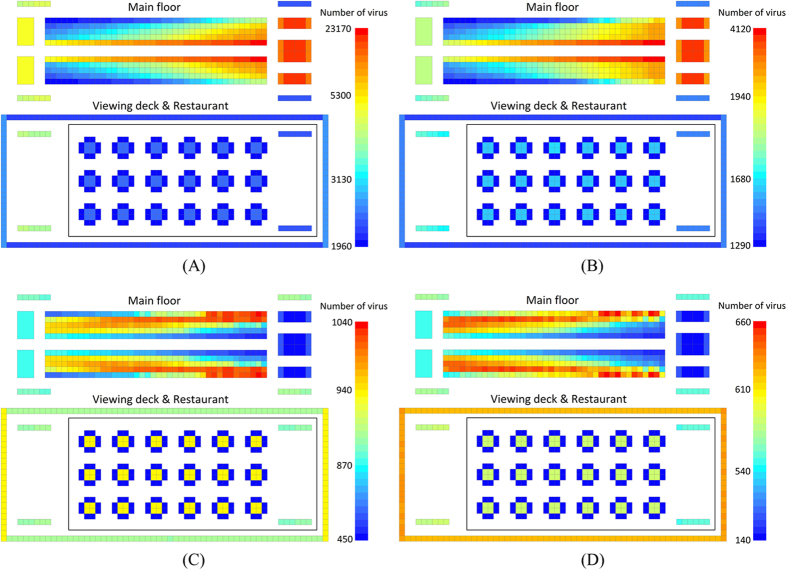
Norovirus propagation on a cruise ship for different probabilities (P) and efficiencies (E) of hand washing (Simulation time: 480 minutes). (**A**) P = E = 0; (**B**) P = E = 30%; (**C**) P = E = 60%; (**D**) P = E = 90%.

Figure 6 shows the norovirus propagation on the cruise ship for different probabilities and efficiencies of hand washing. The bar with different colors represents different numbers of the virus. The total number of viruses dramatically decreases with the increasing probability (P) and efficiency (E) of hand washing. When P and E increased from 30 percent to 60 percent, the virus distribution on seats is seen to be totally different. In addition, when P = E = 0, the virus concentrated on the right seats and the seats near the main aisle. But when P = E = 0.9, the virus concentrated on the left seats and the seats far from the main aisle. If we compare Fig. 6(B) and (C), we can see that with increasing P and E, the impact of going to the toilet is changed from negative to positive. In Fig. 6(B) where door handles are carrying a large number of viruses, it can be seen that the number of viruses on the outer doorknobs is higher than on the inner doorknobs. On the contrary, in Fig. 6(C) the door handles are blue which means they have a low volume of virus. The volume of virus on the inner door handles is even lower. Therefore, the turning point of impact on virus distribution caused by P and E is between 0.3 and 0.6. When P and E increased from 0.6 to 0.9, virus is more concentrated near the wall. At the moment, restrooms appear to repulse viruses.

Comparing the virus distribution on stair railings on cruise ship, the volume of virus on stair railings to the sightseeing deck is always higher than it is on stair railings to the restaurant because the restaurant is visited less frequently. In the restaurant, the relative volume of the virus on dining tables increases with rising P and E. Restrooms have a strong disinfection effect when P and E are high. The volume of the virus on the main floor is reduced, so the relative volume of the virus in the restaurant increased. However, in all situations, since they have a low contact frequency, dining chairs are the only area that always has the lowest volume of virus. The relative volume of the virus on the sightseeing deck increases with increasing P and E.

**Figure 7:**
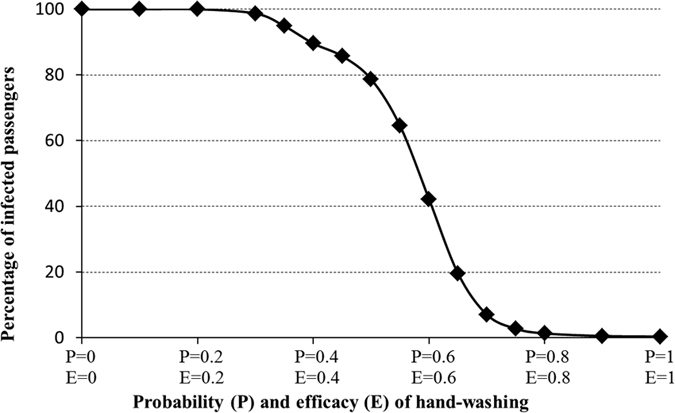
Relationship between percentage of infected passengers and hand washing.

Figure 7 quantifies the relationship between the percentage of infected passengers and hand washing. The percentage of infected passengers decreases with a logistic curve as P and E gradually increase. P and E have little influence on the impact on the percentage of infected passengers when P and E are very low or very high. In the middle of the curve, the impact of P and E is strong. When E = P = 0.8, 1.37 percent of passengers will get infected, which means about 5.5 passengers will change to norovirus-positive from negative. More than 78 percent of passengers will be infected when E = P = 0.5. Through quantitative analysis we show that the sensitive range of P and E is between 0.3 and 0.7. Therefore, controlling P and E to be greater than 0.7 is very critical in the prevention of severe infection.

### Virus propagation with different transfer rates between surfaces

**Figure 8:**
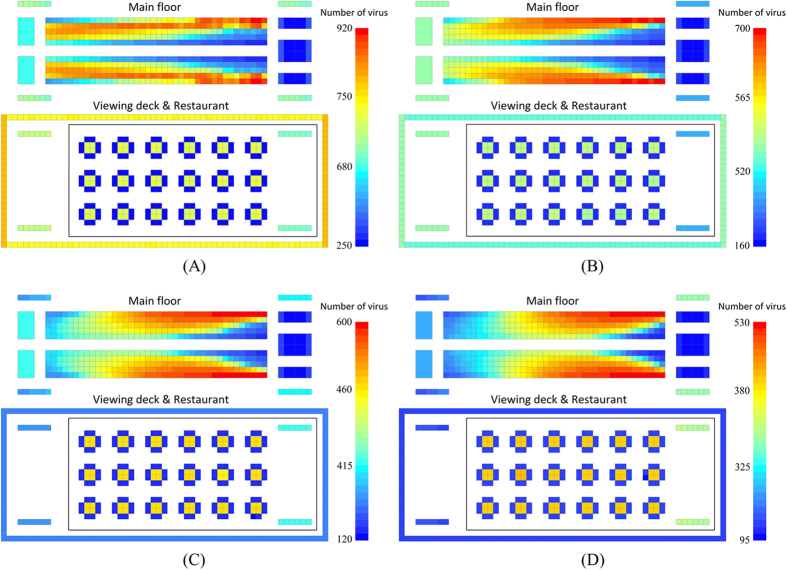
Norovirus propagation on the cruise ship under different transfer rates (RT) between surfaces (Simulation time: 480 minutes). (**A**) RT = 0.1; (**B**) RT = 0.2; (**C**) RT = 0.3; (**D**) RT = 0.4.

Figure 8 shows the virus distribution with different transfer rates (R_T_) between surfaces. With an increasing transfer rate, the area with high-density virus moves to the right. When R_T_ = 0.1, the volume of virus on the seats near the main aisle gradually decreases from left to right. But when R_T_ = 0.4, the middle seats closer to the main aisle have the most virus. Moreover, with increasing R_T_, the concentricity of virus distribution becomes more obvious. As shown in Fig. 8(D), the high-risk areas are close to the wall and close to the restrooms, other than presented in Fig. 8(A). Restrooms are regarded as virus sources during the early stages, but later become disinfection sources because of the high probabilities and efficiencies of hand washing. When the transfer rate is low, because ‘clean passengers’ leaving the restrooms make it difficult to dilute the virus near the restroom, the impact of the restroom on virus distribution is small. If the transfer rate is high, this impact is greater. Combining dilution by retail counters, stair handrails and the sightseeing deck railing, the virus distribution accords with the distribution shown in Fig. 8(D). In reality, the transfer rate is determined by many factors such as the material of the surfaces and personal behavior (contact duration). After confirming the transfer rate, the areas with the highest risk could be predicted. Combined with disinfecting the high-risk areas, the infection rate could be effectively reduced.

**Figure 9:**
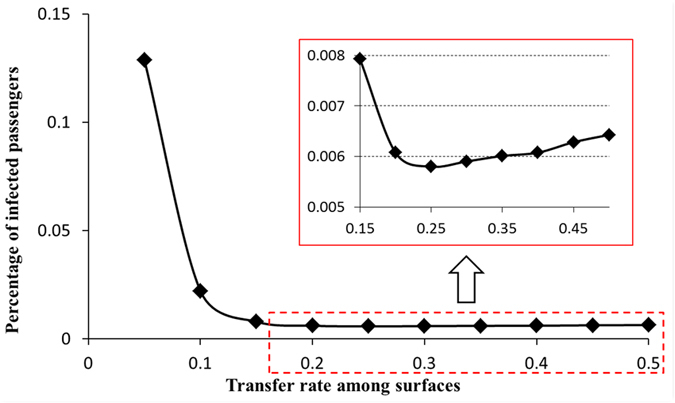
Quantitative analysis of the relationship between the percentage of infected passengers and the transfer rate between surfaces.

Figure 9 quantitatively analyzed the relationship between the infection rate and the transfer rate. Obviously, when R_T_ is less than 0.2, the percentage of infected passengers dramatically decreases with increasing transfer rate. If R_T_ is too low, the high volume of virus carried by some passengers is difficult to dilute via surface contact. However, looking at the red square in Fig. 9, we can see that the infection rate increases slightly with increasing R_T_ when R_T_ is greater than 0.25. For this value of R_T_ the disinfection effect of restrooms is small, and passengers will be rapidly contaminated by the virus from other surfaces. From these results we infer that controlling the transfer rate can effectively prevent passengers from being infected. For example, in this case, the infection risk will reach a minimum when the transfer rate is equal to 0.25. At this time, the infection rate reached to 0.0058, and about 2.32 passengers would be norovirus-positive. When transfer rate reduce to 0.10 from 0.25, the number of norovirus-positive passengers will increase to 8.84.

### Virus propagation with disinfection

**Figure 10:**
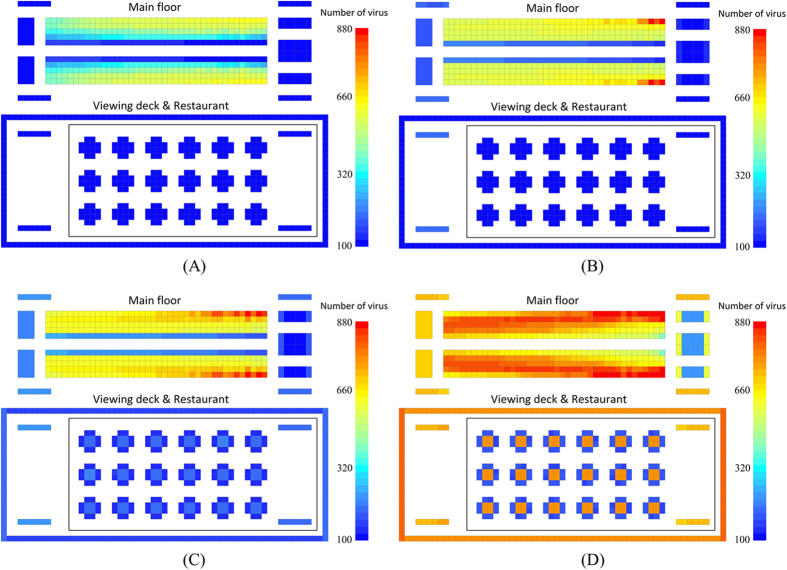
Norovirus propagation on the cruise ship under different intervals of disinfection (ID) (Simulation time: 480 minutes). (**A**) ID = 1 hour; (**B**) ID = 2 hours; (**C**) ID = 4 hours; (**D**) Never.

Figure 10 shows the virus propagation for different disinfection frequencies (once per hour; once every 2 hours; once every 4 hours; never). The total number of virus microorganisms increases with decreasing disinfection frequency. Because of the distance from the virus source, the relative number of virus microorganisms is low in the restaurant and on the sightseeing deck. When the disinfection frequency is once every four hours, the average virus number on every surface in these two functional areas is less than 200. If we compare this with the condition where there is no disinfection (Fig. 10(D)), the total virus number is obviously reduced with disinfection. Dining chairs are difficult to clean, but from Fig. 10, we see that dining chairs always have a low volume of virus whether there is disinfection or not. The main reasons for this are short contact times and the long distance from the virus sources. In contrast to dining chairs, handrails of stairs, handrails on the sightseeing deck, and dining tables have higher contact frequency. For these surfaces, the total virus number could be effectively reduced by regular disinfection.

On the main floor, the functional areas including restrooms, retail counters, and the stairs to the restaurants and the sightseeing deck are disinfected. Because many disinfected surfaces can dilute the virus, when the disinfection frequency is high, the virus is distributed uniformly on the seats that are nonadjacent to the main aisle. As shown in Fig. 10(D), under the condition of no disinfection, virus distribution is V-shaped. The total virus number and the characteristics of the virus distribution can be influenced by regular disinfection. In reality, depending on the nature of the virus, a minimal disinfection frequency could be calculated that would contain the risk.

**Figure 11:**
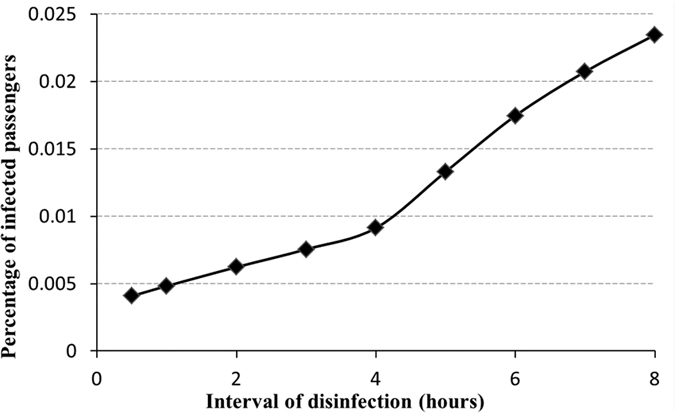
Relationship between the percentage of infected passengers and the interval of disinfection.

Figure 11 quantifies the impact of disinfection frequency on the percentage of infected passengers. The percentage of infected passengers gradually increases with a decreasing disinfection frequency. When I_D_ = 1, the infection rate reached 0.0048, on overage, 1.93 passengers would be norovirus-positive after 8 hours journey on the cruise ship. While I_D_ = 8, the infection rate will increase to 0.023. In Fig. 11, the point when I_D_ = 4 hours is the inflection point. When the interval between disinfections is greater than the inflection point, the efficiency in reducing the number of infected passengers is reduced. On the other hand, efficiency is higher when the interval is shorter than that at the inflection point. From this study, taking into account the layout of the cruise ship and the virus propagation simulation for different disinfection frequencies, some suggestions can be made regarding infectious disease prevention.

**Figure 12:**
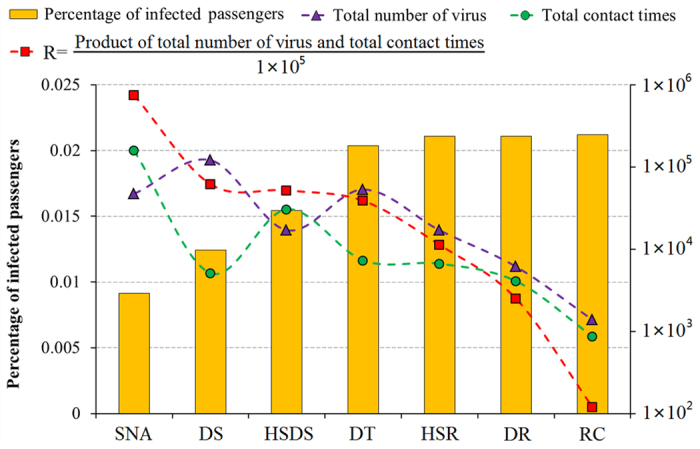
Relationship among percentage of infected passengers, total number of virus and total contact times based on the sensitivity analysis of disinfection of different surfaces. (SNA: seat near the aisle; DS: sightseeing deck; HSDS: handrail of stairs to the sightseeing deck; DT: dining table; HSR: handrail of stairs to the restaurant; DR: doorknob of restroom; RC: Retail counter).

We considered 7 areas for disinfection as described in Fig. 12. The yellow bar in Fig. 12 shows the relationship between the percentage of infected passengers and disinfection of the different surfaces. Disinfection of the seats near the main aisle has the strongest effect on reducing infection. Disinfection on the sightseeing deck and the handrails to the deck ranked second. Disinfection of retail counters, the inner and outer door handles of restrooms, and the handrail to the restaurant was not that useful. In order to clarify the sensitivity of surface disinfection to infection, the total number of virus microorganisms (purple line) and the total contact time (green line) of each functional area are shown in Fig. 12. In this paper, high sensitivity is defined as being when disinfection of a surface is very helpful in reducing the infection rate, while low sensitivity means that disinfection makes no difference. In Fig. 12, as the sensitivity decreases, the total virus number and the contact time shows a decreasing trend. For example, the sensitivities of retail counters and door handles of restrooms are the lowest, and the total virus number and contact times of these are also the lowest. However, the relationship between sensitivity and these two factors is not monotonous. For example, the total virus number is large but the contact times are short for the handrails on the sightseeing deck. Therefore, we use the product of the total virus number and contact times to balance the sensitivity of disinfection surfaces (in order to fit the right Y-axis, we use the parameter R (red line) which equals the product divided by 10^5^ to reflect both total virus number and contact times). With a decreasing sensitivity, R is monotonously decreasing. Therefore, the disinfection effect of surfaces of the different functional areas is related to the product of total virus number and contact times. We should therefore prioritize the disinfection of the surfaces with a high R-value. In this study, the seats near the main aisle and the handrails on the sightseeing deck should be given the most attention.

## Limitations

There are also some limitations of this study. Our study focuses in the computer modelling of the probability of contamination through personal contacts so as to identify potential methods to control infection in actual context (without laboratory support) by the frontline workers (e.g. cruise ship workers) to avoid outbreak. Meanwhile, we will explore the importance of how the use of laboratory investigations (and their indicators) might further support infection control in future studies. In addition, limited passenger’s movement regulation and influencing factors in this study are also constrained to develop effective norovirus propagation measures in cruise ship. The total number on the cruise ship is 400 and simulation time is 8 hours. The results are only suitable for the norovirus propagation in this scale. In addition, all hypotheses from H_1_ to H_11_ are given in the section of passenger behaviors and the results could be referred in these conditions. Based on the computational simulation, the results can partially reflected the conclusion on relationship among norovirus infection and other influencing factors.

## Conclusion

This study focuses on the potential transmission dynamics of contact based infectious diseases. For future investigation, similar models should be built to examine air-borne, food borne as well as vector-borne diseases. In addition, to ensure effective infection control in public locations, other important public health measures such as public risk communication and education of the use of face masks, hand hygiene, proper food safety and sanitation maintenance would be important. Future studies should include how these important dynamics might affect infection control in various settings.

In this paper, using norovirus gastroenteritis as an example, we study the contact infection of an infectious disease on a cruise ship. We considered six functional areas: seats; restrooms; retail counters; stair handrails; restaurant; and sightseeing deck railings, as well as eight contact surfaces. Combining the influencing factors of personal behavior, virus transfer between surfaces, virus reproduction, passenger infection, and disinfection, we simulated infectious disease propagation on a cruise ship.

In the control group, passengers far away from the restrooms have a higher infection rate than those near the restrooms. In this case, virus distribution across the seating area is in the shape of a V, with the seats nearer the main aisle being cleaner than those further away. The volume of virus microorganisms in the restaurant is low because of infrequent contact, and dining chairs are cleaner than dining tables. There are more viruses on the sightseeing deck, with the average number of viruses on the shorter handrails being higher than found on the longer handrails.

The probability and efficiency of hand washing is the most important factor in determining the number and distribution of the virus. On the main floor, the left side is cleaner than the right side and the seats nearer the main aisle have more viruses. With an increasing probability and efficiency of hand washing, the distribution of the virus changes, and volume of virus microorganisms in the aisle and on the right side decreases. Through quantitative analysis we find that the percentage of infected passengers decreases rapidly when the probability and efficiency of hand washing increases from 0.3 to 0.7. Therefore, keeping the probability and efficiency of hand washing over 70 percent is very important to prevent infection.

The virus transfer rate between surfaces decides the distribution and concentration of the virus. High transfer rate led to high concentricity. With a low transfer rate, virus distribution was shaped like a V, and the number of virus microorganisms in the high-density areas was greater than 900 per grid. When the transfer rate rose to 0.4, the viruses were densely distributed near the restrooms and on the walls. The number of virus microorganisms in the high-density areas was approximately 500 per grid. Through quantitative analysis we found that the infection rate was lowest when the transfer rate was 0.25. If contact surfaces of the densely populated areas on board ship were designed to reduce roughness and control relative factors to keep the transfer rate at 0.25, this would help prevent virus propagation and reduce the infection rate.

Through simulation we can see that regular disinfection of seats near the main aisle, door handles of the restrooms, handrails of stairs and on the sightseeing deck can effectively reduce the total number of virus microorganisms. The average number of virus microorganisms per grid on the sightseeing deck and in the restaurant is around 100 when the disinfection frequency is once every 2 hours. When the disinfection frequency increased to once per hour, the grid with the highest risk had only 600 virus microorganisms. Comparing this with the condition where there is no disinfection (majority of seats have 800 virus microorganisms), the total number of virus microorganisms is dramatically reduced. Based on quantitative analysis, a disinfection frequency of once every 4 hours is an inflection point. Continuing to increase the disinfection frequency will reduce the efficiency of its impact on infection prevention. Finally, we found that disinfecting the surfaces with the highest total virus number and longest contact times is more useful in reducing the percentage of infected passengers.

All obtained results can provide some scientific basis for contact infection of infectious diseases in densely-populated places. The results also provide the basis for suggestions on how to control and prevent infectious disease.

## Additional Information

**How to cite this article**: Zhang, N. *et al*. Contact infection of infectious disease onboard a cruise ship. *Sci. Rep.* **6**, 38790; doi: 10.1038/srep38790 (2016).

**Publisher's note:** Springer Nature remains neutral with regard to jurisdictional claims in published maps and institutional affiliations.

## Figures and Tables

**Table 1:** The value of each factor in the control group.

Parameter	Value	Parameter	Value
Probability of hand washing	80%	Probability of using the toilet per minute	1/160
Efficiency of hand washing	72.5%	Probability of going to retail per minute	1/180
Time of staying at retail counter	1 min	Virus reproduction rate per minute	1.0138
Time for having a meal	25 min	Time of staying at the sightseeing deck	12 min
Initial number of virus microorganisms	3000	Number of virus microorganisms leading to infection	1000
Probability of going to restaurant per minute	1/80	Virus transfer rate from high-density surfaces to low-density surfaces	10%
Probability of going to the sightseeing deck per minute	1/120	Probability of passengers holding the seats when they move	100%

## References

[b1] Buckley R., Gretzel U. & Scott D. Tourism megatrends. Tourism Recreation Research, 40, 59–70 (2015).

[b2] Diedrich A. Cruise ship tourism in Belize: The implications of developing cruise ship tourism in an ecotourism destination. Ocean & Coastal Management 53, 234–244 (2010).

[b3] Cruise Lines International Association. 2010 CLIA cruise market overview: statistical cruise industry data through 2009. Available at: http://www2.cruising.org/Press/overview2010/. (Accessed 2010 Dec 6).

[b4] Ward K. A., Paul A., Mcanulty J. M., Iwasenko J. M. & Dwyer D. E. Outbreaks of pandemic (H1N1) 2009 and seasonal influenza A (H3N2) on cruise ship. Emerging Infectious Diseases 16, 1731–1737 (2010). 21029531 10.3201/eid1611.100477 PMC3294517

[b5] Kiren M. . Measles, Rubella, and Varicella Among the Crew of a Cruise Ship Sailing From Florida, United States, 2006. Journal of Travel Medicine 19, 233–237 (2012). 22776384 10.1111/j.1708-8305.2012.00620.x

[b6] Cramer E. H. . Epidemiology of gastroenteritis on cruise ships, 2001–2004. American Journal of Preventive Medicine 30, 252–257 (2006). 16476642 10.1016/j.amepre.2005.10.027

[b7] Krzysztof K. & Katarzyna P. Malaria–a disease of travellers. Przegla̧d Epidemiologiczny 66, 591–597 (2012). 23484386

[b8] Isakbaeva E. T. . Norovirus transmission on cruise ship. Emerging Infectious Disease 11, 154–158 (2005). 10.3201/eid1101.040434 PMC3294347 15705344

[b9] Lee H. & Ko G. P. Antiviral effect of vitamin A on norovirus infection via modulation of the gut microbiome. Scientific Reports, 6, 25835 (2016). 27180604 10.1038/srep25835 PMC4867650

[b10] Zhang X. F. . An outbreak caused by GII.17 norovirus with a wide spectrum of HBGA-associated susceptibility. Scientific Reports 5, 17687 (2015). 26639056 10.1038/srep17687 PMC4671059

[b11] Xuan W. . An outbreak of multiple norovirus strains on a cruise ship in China, 2014. Journal of Applied Microbiology 120, 226–233 (2016). 26481457 10.1111/jam.12978

[b12] Morillo S. G., Luchs A. & Cilli A. Rapid detection of norovirus in naturally contaminated food: foodborne gastroenteritis outbreak on a cruise ship in Brazil, 2010. Food & Environmental Virology 4, 124–129 (2012). 23412839 10.1007/s12560-012-9085-x

[b13] Chimonas M. R. . Passenger behaviors associated with norovirus infection on board a cruise ship—Alaska, May to June 2004. Journal of Travel Medicine 15, 177–183 (2008). 18494695 10.1111/j.1708-8305.2008.00200.x

[b14] Vivancos R. . Norovirus outbreak in a cruise ship sailing around the British Isles: Investigation and multi-agency management of an international outbreak. Journal of Infection 60, 478–485 (2010). 20359496 10.1016/j.jinf.2010.03.018

[b15] Freedman S., Winn R., Wolstenholme P., Jones G. & Firmin S. Pump Station Refurbishment Designed for Compatibility with Adjacent Condominium, Hotel and Marine Terminal Development Projects in Portland, Maine. Proceedings of the Water Environment Federation 2008, 836–843 (2008).

[b16] Qin X. H. Investigation report of consumption behavior in tourism marketing in Zhengzhou city. China Statistics 9, 60–61 (In Chinese) (2011).

[b17] Thornley C. N., Emslie N. A., Sprott T. W., Greening G. E. & Rapana J. P. Recurring norovirus transmission on an airplane. Clinical Infectious Diseases 521–526 (2011). 21836128 10.1093/cid/cir465

[b18] Weber D. J., Rutala W. A., Miller M. B., Huslage K. & Sickbert-Bennett E. Role of hospital surfaces in the transmission of emerging health care-associated pathogens: norovirus, Clostridium difficile, and Acinetobacter species. American Journal of Infection Control 38, S25–S33 (2010). 20569853 10.1016/j.ajic.2010.04.196

[b19] Ruan F. . Risk factors for hand, foot, and mouth disease and herpangina and the preventive effect of hand-washing. Pediatrics 127, e898–e904 (2011). 21422083 10.1542/peds.2010-1497

[b20] Celik S. & Koçaşli S. Hygienic hand washing among nursing students in Turkey. Applied Nursing Research Anr 21, 207–11 (2008). 18995162 10.1016/j.apnr.2006.12.001

[b21] Duggan J. M., Hensley S., Khuder S., Papadimos T. J. & Jacobs L. Inverse correlation between level of professional education and rate of handwashing compliance in a teaching hospital. Infection Control & Hospital Epidemiology 29, 534–538 (2008). 18476776 10.1086/588164

[b22] Shu B. . Investigation on hand washing behavior of residents in Guangzhou. South China Journal of Preventive Medicine 5, 57–58 (In Chinese) (2009).

[b23] Ansari S. A., Sattar S. A., Springthorpe V. S., Wells G. A. & Tostowaryk W. *In vivo* protocol for testing efficacy of hand washing agents against viruses and bacteria: experiments with rotavirus and Escherichia coli. Applied & Environmental Microbiology 55, 3113–3118 (1989). 2559658 10.1128/aem.55.12.3113-3118.1989 PMC203232

[b24] Murphy L., Mascardo G. & Benckendorff P. Exploring word-of-mouth influences on travel decisions: friends and relatives vs. other travellers. International Ijc 31, 517–527 (2007).

[b25] Courvoisier D. S., Eid M. & Lischetzke T. Compliance to a cell phone-based ecological momentary assessment study: the effect of time and personality characteristics. Psychological assessment 24, 713–720 (2012). 22250597 10.1037/a0026733

[b26] Shiplee V. & Johnson A. Control of norovirus on a cruise ship. Food Science & Technology 28–29 (2014).

[b27] Díaz C., Schilardi P. & de Mele M. F. L. Influence of surface sub-micropattern on the adhesion of pioneer bacteria on metals. Artificial Organs 32, 292–298 (2008). 18370943 10.1111/j.1525-1594.2008.00545.x

[b28] Zobell C. E. & Cobet A. B. Growth, reproduction, and death rates of Escherichia coli at increased hydrostatic pressures. Journal of Bacteriology 84, 1228–1236 (1962). 14003850 10.1128/jb.84.6.1228-1236.1962 PMC278050

[b29] Fuxman Y. L. Reproduction rate, feeding process, and liebig limitations in cell populations—II. Bulletin of Mathematical Biology 57, 749–782 (1995). 7606224 10.1007/BF02461850

[b30] Dolin R. Noroviruses–challenges to control. New England Journal of Medicine 357, 1072–1073 (2007). 17855667 10.1056/NEJMp078050

[b31] Carling P. C., Bruno-Murtha L. A. & Griffiths J. K. Cruise ship environmental hygiene and the risk of norovirus infection outbreaks: an objective assessment of 56 vessels over 3 years. Clinical Infectious Diseases 49, 1312–1317 (2009). 19814610 10.1086/606058

[b32] Cramer E. H. . Outbreaks of gastroenteritis associated with noroviruses on cruise ships- United States, 2002. Morbidity and Mortality Weekly Report 51, 1112–1115 (2002). 12530708

[b33] Chimonas M. A. R. . Passenger behaviors associated with norovirus infection on board a cruise ship- Alaska, May to June 2004. Journal of Travel Medicine 15, 177–183 (2008). 18494695 10.1111/j.1708-8305.2008.00200.x

